# On Quinoidine in the Treatment of Intermittent Fever

**Published:** 1855-05

**Authors:** J. DaCosta


### *On Quinoidine in the Treatment of Intermittent Fever.*

By J. DaCosta, M. D.

Amongst the preparations of bark that have occasionally been used for the cure of intermittent fever, we find quinoidine, or the so-called "amorphous quinia" mentioned.* Yet, although the price of this article is considerably less than that of the favored salts of its twin-alkaloid, quinia, it has met with but little medical notice or employ; the publication, therefore, of upwards of fifty cases treated successfully with quinoidine, may prove, the writer hopes, not uninteresting nor valueless to some of the readers of this journal.

Chinoidinia, or "quinoidine," was first brought into notice by the supposed discovery of Sertürner of a new alkaloid, obtained by treating acidulous extracts of cinchona bark by alkalies. This alkaloid he found associated with quinia and cinchonia, and he procured it in large quantities from the resinous substance remaining after the crystallization of sulphate of quinia, which

* To guard against error, it may be well to state that quinoidine is an entirely different substance from the quinidine, the sulphate of which has been recently discovered to possess antiperiodic properties. (See Med. Examiner, Vol. x.)

mass itself had previously been employed as an antiperiodic in this city, under the name of "extract of quinine." (See U. S. Dispensatory, 10th edition, p. 1171.)

Several chemists, induced by Sertürner's description of the new alkaloid, endeavored to procure it in its isolated state, as he stated he had done, but they were not successful; and the opinion, strengthened by the experiments of MM. Delondre, Henry and Geiger, soon gained ground, that the supposed new alkaloid discovered in the mother liquor after the crystallization of sulphate of quinia, was in reality quinia and cinchonia, in union with a resinous substance, which impeded their crystallization. Liebig,* who analyzed several samples of quinoidine, found a considerable proportion of it to possess the chemical characters of a true organic base, and to have the same atomic weight and composition as ordinary quinine, to which he conceived it to bear the same relation that uncrystalline sugar does to crystalline. The resinous alkaloid obtained by evaporating an ethereal solution of the flakes, (precipitated from an acid solution by ammonia, and alkaline carbonates,) he termed, therefore, "amorphous quinia," and this view of the nature of quinoidine seemed to be confirmed by the statement of Winkler, that ordinary quinia may be rendered amorphous by the action of acids, and by the experiments of Roder, (Chem. Gaz., 1848), who obtained from the amorphous quinia, quinia in a crystalline state. Shortly after the publication of Liebig's analysis, a patent, probably through his influence, was granted to Mr. Bullock, of London, for the manufacture of the "amorphous quinia," or this new extract from the quinoidine. The process, however, by which the latter was thus entirely deprived of the resin, and reduced to "amorphous quinia," rendered this an extremely expensive preparation, nor was it found to differ from the ordinary quinoidine of commerce, except in its being more highly purified.† Later analyses than those of Liebig have tended to confirm the fact that quinoidine is in reality the amorphous alkaloid quinia, in union with a resinous substance, and capable of forming salts and acids; yet there are some chemists who consider it to be a

* London Lancet, May 23, 1846.

† See Mr. Redwood's paper, Pharmaceutical Journal, vol. vi. p. 131.

compound of quinia, cinchonia, and quinidia, and others again who still regard its true composition as unascertained.*

Quinoidine sufficiently pure for medicinal purposes, is prepared in this city by several eminent chemists, (especially, we believe, by Messrs. Powers & Weightman,) by precipitation, by means of alkalies, from the acidulous solution of the residue left after the crystallization of the sulphate of quinia. The precipitate thus formed is dissolved in alcohol, and from this solution, subsequently, the quinoidine is obtained. The amount of this alkaloid furnished by different barks varies. Thus, the red bark, which contains less quinia than the Calisaya, yields a larger amount of quinoidine. Mr. Weightman recently informed the writer that the ordinary Calisaya bark furnishes, in proportion to every 100 parts of quinia, only about 10 to 20 of quinoidine, while the New Granada barks yield in proportion to the quinine they contain, about 40 per cent. of the quinoidine.

Quinoidine, when pure, is soluble in alcohol and sulphuric acid, from which it may again be precipitated without loss of weight. Its color is that of an ordinary extract; it is almost entirely devoid of taste. It has been occasionally medicinally employed, especially in some parts of Continental Europe, and enjoyed a high reputation as an antiperiodic in an epidemic that occurred near Berlin, (see London Lancet, 1846,) so much so, that the peasants came from fifty miles distant to procure certain "fever drops," which consisted of the medicine in question dissolved in sulphuric acid. In this country quinoidine, owing, perhaps, to its liability to be adulterated, has been but little employed, excepting by the venders of quack medicines, whose nostrums, in many instances, owe their efficacy to the quinoidine they contain.

The therapeutical effect of quinoidine, as far as the writer has observed them, are very nearly those of the ordinary sulphate of quinia. In doses two thirds larger than those of the latter, he succeeded perfectly in checking intermittent fever; but it was never noted to give rise to the headache, nor the ringing and buzzing in the ears, nor to the sickness at the stomach, which so frequently attend the administration of the sulphate of quinia, or of the other ordinary preparations of bark. It was

*Ibid, vol. xi. 1850.

sometimes given in doses of 40 grains, without the slightest inconvenience to the patient resulting from it, and in no case, with the exception of one subjoined below (see case 32,) did it fail in checking the periodical returns of the paroxysms. In a few instances, indeed, it proved successful when sulphate of quinia had been previously administered without result. As a tonic, it seems to possess the same advantages as any of the other preparations of bark. Age does not constitute an objection to its use, for it was employed with equal advantage in the very young and the very old.*

The patients for whom quinoidine was prescribed, were mostly Irish of the lower classes, who applied for advice to the Moyamensing House of Industry. In many, the intermittent fever had lasted for some length of time; it was mostly of the quotidian type. No cathartic was ordered previous to the administration of the medicine, which was given in pills of two grains each, or else dissolved in dilute aromatic sulphuric acid. The average dose required to arrest the chill in an adult seemed to be about 20 grains, six grains of which were, as a general rule, ordered shortly before the expected paroxysm, whilst the rest was taken during the intermission. If the bowels were constipated, a little of the extract of colycinth was with advantage joined to the quinoidine. After the chills were checked, the remedy was in some cases continued, in smaller doses; yet as patients, when relieved, are not always willing to return to a public institution, it was sometimes impossible to persevere with the treatment, and the disease then generally reappeared after the lapse of a week or two. No other medicine was administered in any of the cases noted, unless specially stated in the subjoined table.

* In proof of the efficacy of this medicine, the writer is happy to be able to add the testimony of one of the gentlemen connected with the chemical works of Messrs. Powers & Weightman on the falls of the Schuylkill. He informed him, that in all the cases of intermittent fever which occur there among the workmen and their families, quinoidine had been used with perfect success, and that the effect was always permanent, which could be easily ascertained, as most of them were under his daily inspection. The medicine was even deemed more efficacious than the other preparations of bark.

| | Name. | Age | Type of Fever. | Date when first seen. | Dose of medicine which arrested chill. | Return of disease. | Remarks. |
|---|---|---|---|---|---|---|---|
| 1 | Benjamin Noble | 14 | quotidian | Sept. 6 | 12 grs. | Sept. 22 | Dose was then repeated; no return of chill since. |
| 2 | William Young | 38 | tertian | " 14 | 22 " | " 20 | Had a slight chill on 20th, for which 20 grs. were given. March 1st, saw him with intermittent fever, which he had contracted from fresh exposure; yielded again to 20 grs. of quinoidine. |
| 3 | William Auscher | 38 | quotidian | " 18 | 20 " | None. | |
| 4 | W. Hall | 29 | tertian | " 18 | 20 " | " | The first paroxysm was modified, but not entirely arrested by 20 grs., as he had a chill on the 22d; prescription repeated; no return since. |
| 5 | Harriet Keller | 15 | quotidian | " 18 | 26 " | " | |
| 6 | John Nesbit | 27 | tertian | " 19 | 30 " | " | Disease of long standing; 30 grs. arrested paroxysm on expected day. Slight chill a few days afterwards; twenty grs. administered, since which time no return. |
| 7 | Eliza Doheny | 12 | quotidian | " 20 | 20 " | " | |
| 8 | John Finley | 30 | quotidian | " 20 | 20 " | " | |
| 9 | Isaac Burns | 50 | tertian | " 22 | 20 " | " | |
| 10 | Augustus Henry | | tertian | " 22 | 20 " | " | |
| 11 | Charles Gowin | 5 | tertian | " 24 | 10 " | " | |
| 12 | T. Erny | 25 | tertian | " 26 | 20 " | " | |
| 13 | Barnet Henely | 35 | quotidian | " 27 | 30 " | " | Pills of quinoidine were given as a tonic for some time after chill was arrested. |
| 14 | Margaret Vaughen | 21 | quotidian | " 29 | 20 " | " | |
| 15 | W. Thompson | 40 | quotidian | Oct. 3 | 40 " | " | |
| 16 | William Bacon | 35 | quotidian | " 3 | 20 " | " | |
| 17 | Louisa Thompson | 55 | quotidian | " 6 | 20 " | " | |
| 18 | Benjamin Buchner | 24 | quotidian | " 18 | 40 " | " | In him the disease had been of long duration; he had previously taken sulphate of quinia. |
| 19 | Patrick McElham | 20 | quotidian | Nov. 8 | 20 " | " | |
| 20 | Patrick Smith | 22 | quotidian | " 8 | 20 " | " | |
| 21 | M. Gibson | 20 | quotidian | " 12 | 20 " | " | |
| 22 | Michael Brown | 17 | tertian | " 12 | 20 " | Nov. 26 | The chills returned subsequently several times at intervals of two weeks, but always yielded to 20 grs. of quinoidine. Since 29th of Jan. he has taken quinoidine grs. 2 t. d. as a tonic. No return of chill since. |
| 23 | Edward Coroll | 28 | tertian | " 13 | 16 " | None. | This patient had, on the 11th, before I saw him, taken 20 grs. of quinoidine, which had not arrested the chill. |
| 24 | John Cushen | 23 | tertian | " 13 | 20 " | " | Medicine was repeated on the 15th; no return. |
| 25 | John McCarty | 23 | quotidian | " 11 | 20 " | Nov. 18 | On Nov. 11th he had a slight chill, for which he took 20 grs. more of quinoidine. Since that time no return. |
| 26 | Thomas Heley | 28 | quotidian | " 15 | 20 " | None. | |

| | Name. | Age | Type of Fever. | Date when first seen. | Dose of medicine which arrested chill. | Return of disease. | Remarks. |
|---|---|---|---|---|---|---|---|
| 27 | Vm. Fitzgerald | 24 | tertian | Nov. 15 | 20 grs. | Jan. 25 | This patient continued taking quinoidine for about a week after arrest of chill; on the 25th of Jan. had a return of the disease, which yielded, however, readily to 20 grs. of quinoidine; since then no return. |
| 28 | Jane Ann Kennedy | 17 | quotidian | " 18 | 16 " | None. | Medicine was repeated on the 19th, and continued until the 26th. |
| 29 | Daniel Mack | 13 | quotidian | " 20 | 12 " | " | |
| 30 | Ann Cumming | 44 | tertian | " 22 | 16 " | " | |
| 31 | Ann Eige | 5 | quotidian | " 23 | 10 " | Dec. 20 | The 10 grs. checked the chills, but they returned a few days afterwards, yielding, however, again to 10 grs. of quinoidine. On Dec. 20, chills returned. The patient was directed to take 4 grs. of sulphate of quinia daily for two weeks. On the 17th of January saw the patient. The chills had not been stopped, but had re-occurred from time to time. Ordered 40 grs. of quinoidine in 20 pills, 4 daily. After medicine had been taken for 3 days, chills stopped; no return since. |
| 32 | Therese Eige | 40 | tertian | " 25 | 20 " | " 7 | This patient had had, when first seen, chills for 8 or 9 months. On Nov. 25, 10 grs. of sulphate of cinchona and 10 of sulph. of quinia were administered, which antiperiodics were continued in smaller doses until Dec. 7. They had then partly, but not entirely broken up the intermittent fever. On Dec. 7th chills returned more violently: 20 grs. of quinoidine stopped the expected paroxysm on the 9th, but although a tonic treatment was adopted, the chills returned on the 21st; 24 grains of sulphate of quinia arrested them for a few days, but they again returned Jan. 27; 20grs. of quinoidine then prescribed; the patient continuing the medicine remained exempt until Feb. 16; chills then reappeared, although quinoidine was being largely administered. Feb. 24 she was placed on 20 grs. of sulph. of quinia daily, with bark after the chills had been arrested, as a tonic. No return of chill since 26th of Feb. |
| 33 | Eliza Holloway | 20 | tertian | " 28 | 20 " | None. | |
| 34 | John Eige | 18 | tertian | " 31 | 20 " | " | |
| 35 | John Kelly | 45 | tertian | Dec. 2 | 30 " | " | |

| | Name. | Age | Type of Fever. | Date when first seen. | Dose of medicine which arrested chill. | Return of disease. | REMARKS. |
|---|---|---|---|---|---|---|---|
| 36 | Edward Hart | 10 | tertian | Dec. 11 | 30 grs. | None | Intermittent fever had lasted since August. |
| 37 | Edward Heinson | 20 | tertian | Jan. 6 | 20 " | " | |
| 38 | William Young | 3 | tertian | " 20 | 30 " | Feb. 13 | Dose was then repeated; no return since. |
| 39 | D. Smith | 37 | tertian | " 22 | 20 " | None. | |
| 40 | Will. Fitzger | 24 | quotidian | " 25 | 20 " | " | |
| 41 | James Wilson | 24 | quotidian | " 26 | 20 " | " | |
| 42 | John Dodd | 48 | quotidian | Feb. 22 | 20 " | " | |
| 43 | Robert Wyley | 39 | quotidian | " 23 | 20 " | " | |
| 44 | Thomas Dougherty | 25 | tertian | " 23 | 20 " | Mar. 7 | Dose repeated on the 8th of March; no return since. |
| 45 | Michael Dougherty | 41 | tertian | " 23 | 20 " | None. | |
| 46 | Patrick Mullish | 25 | quotidian | March 2 | 20 " | " | |
| 47 | Francis Jordan | 11 | quotidian | " 7 | 20 " | " | |
| 48 | Mary Hagan | 39 | tertian | " 9 | 30 " | Mar. 28 | The disease in this patient was contracted in Aug., 1854, on the banks of the Ohio river. She had been taking in large quantities sul. of quinia, which arrested the chills for a short time, but did not prevent their recurrence; 30 grs. of quinoidine arrested the chill on the 9th of March, and prevented the paroxysm until the 28th. She has been since continuing quinoidine, and has had as yet no return of the chill. |
| 49 | Sarah Hagan | 16 | quotidian | " 10 | 20 " | None. | |
| 50 | Mary Argus | 20 | double quotidian | " 10 | 140 " | Mar. 13 | 20 grs. were repeated on the 13th and 14th; yet chills every day until 17th. From that time no chill; medicine has been continued. |
| 51 | M. Murphy | 24 | quotidian | " 15 | 40 " | None. | The disease in this instance was of 6 months' standing; 40 grs. arrested the chill on the 16th; medicine since continued; no return of chills. |
| 52 | Susan Hoggarty | 40 | quotidian | " 22 | 30 " | " | |
| 53 | Mariol Brooks | 21 | tertian | " 22 | 20 " | Mar. 20 | Commenced as quotidian in August; for the last 2 months assumed the tertian type. Quinia had been taken repeatedly, (although not in large doses) without producing much effect. Had a slight chill on the 23d; quinoidine has been continued in smaller doses since; no return of disease up to April 17th. |

The writer might add notes of several more patients who were treated by quinoidine with equal success; he judges, however, the publication of the above cases to be sufficient to prove the efficacy of the remedy. Of the 53 cases cited, in many of whom the disease was of long standing, the chills were arrested in 49

cases by the first administration of the medicine; only 4 required a repetition of the dose. In 10 cases the disease returned, which, although it may seem a large proportion of relapses, is not in reality so, when we consider the well known tendency of intermittent fever to return, and the fact that, in many of the cases, no medicine, for reasons above stated, was given after the first arrest of the chill. In conclusion, the writer can state as his honest belief, that quinoidine possesses antiperiodic qualities which, if not superior, are certainly not inferior to the sulphate of quinia or cinchonia, whilst he thinks it preferable to these, from the absence of bitter taste, from its being less liable to affect the head or the stomach of the patient, and from its comparatively low price.*

*About one-seventh of the price of the sulphate of quinia.

